# Effect of Formalin Fixation on Surgical Margins in Breast Cancer Surgical Specimen

**DOI:** 10.1155/2014/121838

**Published:** 2014-10-02

**Authors:** Masooma Zaidi, Shaista Khan, Najiha Bilal Farooqi, Kashif Abbas, Romana Idrees

**Affiliations:** ^1^Queen Alexandra Hospital, Southwick Hill Road, Portsmouth Hampshire PO6 3LY, UK; ^2^The Aga Khan University Hospital, Stadium Road, Karachi 74800, Pakistan; ^3^Islam Medical and Dental College, Pasrur Road, Sialkot, Pakistan

## Abstract

Margin analysis in breast surgery is an important predictor of local recurrence and can have vital impact on the postoperative treatment planning.* Objective*. The aim was to assess the mean reduction in the closest tumor-free surgical margin in millimeters of breast cancer specimens following formalin fixation.* Materials and Methods*. We conducted a cross-sectional study at the Aga Khan University Hospital from March 30, 2010 to January 20, 2011. One hundred consecutive breast tumour surgical specimens which had macroscopically visible tumour were included. The cancer type included both in situ and invasive cancers. Excluded were the patients who had previous surgery or systemic/radiation therapy. The closest tumor-free margin was recorded and compared with the margin after formalin fixation. *P* value of <0.05 was considered significant.* Results*. The mean age of our 100 patients was 53 years with the majority of the patients having undergone mastectomy for predominantly invasive ductal carcinoma. Following formalin fixation, the mean reduction of the closest tumor-free margin was noted as 2.14 mm which was found statistically significant.* Conclusion*. Considerable shrinkage of tumor-free surgical margins of breast cancer specimen was noted after formalin fixation. This inference can have implications on the postoperative management plan.

## 1. Introduction

Breast cancer is the most common cancer in women worldwide [1–3]. Approximately 1 million cases are diagnosed each year of which 42% are from the developing world [1]. Pakistan shows the highest reported incidence of breast cancer from Asia, as per the available literature [4, 5]. It is the most common malignancy found among women in Pakistan [6, 7]. Surgery is an essential component of the management of breast cancer. There has been a recent paradigm shift in the surgical management of breast cancer from radical surgery to breast conserving therapy (BCT) with better cosmetic results. For early invasive cancer, survival rates, after BCT, are comparable to those obtained after radical mastectomy [8]. In BCT patients, however, there is a risk of local recurrence. Among the predictors of local recurrence, surgical margin analysis has proven to be the strongest [8–11]. Despite a few recent guidelines [12], no clear consensus has been reached globally regarding the surgical margin analysis [10, 11, 13].

Specimen shrinkage during tissue processing is well known. However, few studies support this for breast specimen [14–16]. Results from a recent study actually contradicted this shrinkage phenomenon for breast tissue [17]. This prompted us to conduct a cross-sectional study on breast cancer specimen, to find out the effect of formalin on the closest tumor-free surgical margin.

## 2. Materials and Methods

The study was carried out at a tertiary care hospital over a period of ten months. We carried out a cross-sectional study on a hundred breast specimens. Nonprobability consecutive sampling technique was used for data collection. Our inclusion criteria involved all patients with histopathologically proven breast cancer undergoing surgery as the primary treatment modality. These patients were undergoing different surgical procedures, namely, wide local excisions, quadrantectomies, and all types of mastectomy (including simple mastectomy and modified radical mastectomy). The types of cancer included were invasive cancers comprising ductal, lobular, metaplastic, medullary, papillary, and ductal cancers with neuroendocrine differentiation. In addition, noninvasive cancers, including ductal carcinoma in situ and noninvasive papillary carcinoma, which could be macroscopically analyzed, were also included. We excluded male patients with breast cancer. Patients who had previously undergone chemotherapy or radiation before the operation were excluded as well. An informed consent was obtained from eligible patients before specimen collection. The specimen was collected from the Operation Theatre and was handed over to the pathologist. A questionnaire, prepared after thorough literature search, was used to record the surgical margins and additional information such as age, stage of the disease, type of surgery, and type and grade of tumor. The breast specimens were delivered fresh without immersion in formalin to the pathologist, who then prepared them. Wide local specimens were sliced into 3-4 mm slices. Mastectomy specimens were cut longitudinally into 2 cm thick slices [18]. All specimen margins were measured on unpreserved tissue. Specimens were subsequently immersed in the 10% buffered formalin overnight. The corresponding measurements were recorded the next morning by the same pathologist. Data was analyzed using SPSS version 16.0. Continuous demographic variable, that is, age, was described as mean ± standard deviation and categorical variables, namely, tumor grade, stage of the disease, type of surgery, final tumor type, and tumor grades, were described as percentages. The measurements of the closest tumor-free surgical margins in relation to specimen fixation were compared by paired *t*-test. Using tumor-free surgical margin as the outcome of interest, stratification was used to assess the impact of variables described in the questionnaire, namely, age, tumor stage, tumor type, and tumor grade. A *P* value of ≤0.05 was considered significant.

## 3. Results

The mean age of the sample size came out to be 53 years with a standard deviation of 13 years. The patient population in the sample size predominantly underwent mastectomy, including modified radical mastectomy and simple mastectomy. Breast conservation surgery, that is, wide local excision and quadrantectomy, was done in 10% (Table 1).

Ninety-two patients in the study population were found to have invasive cancer, whereas carcinoma in situ which was exclusively ductal carcinoma in situ (DCIS) was found in the remaining eight patients (Table 1).

The outcome variable was the closest tumor-free margin reduction after fixation of the specimen with formalin. As shown in Table 2, after applying paired sample *t*-test, the mean reduction in margin was found to be 2.14 mm which was statistically significant (*P* value ≤0.0001). Using the reduction in tumor-free margin after formalin fixation as our outcome of interest, stratification was used to assess the impact of variables described in the questionnaire, namely, age, tumor stage, tumor type, and tumor grade; mean margin reduction was also evaluated for each of these variable groups and was found to be statistically insignificant; that is, *P* value >0.05 for all (Table 3).

## 4. Discussion

Breast cancer is the most common malignancy in females showing an increasing incidence [4]. Pakistan is considered one of the high risk regions as far as breast cancer is concerned [2]. Current treatment strategies take into account properties of the individual patient's tumor biology, as well as the size and location of tumor, to guide treatment [19, 20]. The treatment options include both surgical resection for local disease and medical therapy for systemic disease. Surgical resection, being the mainstay of treatment, has come a long way from the time of radical mastectomy to the breast conservation therapy being extensively practiced nowadays [20].

Due to the lack of screening program and population awareness in Pakistan, most of the patients would present at a younger age with a more advanced disease such as stage 3 or stage 4 and are lymph node positive [5, 21]. This is one of the reasons behind most of the patients in our study having mastectomy rather than BCT. Also, at the time period of this study, we were in process of shifting to BCT with a cautious approach towards neoadjuvant chemotherapy to make breast conservation possible.

With the increasing number of breast conservation surgeries being performed worldwide for breast cancers, the analysis of surgical margins has gained an important value in determining the outcome of treatment. Many a time, it has been categorized as the most important factor for predicting local recurrence after surgery [8–11]. Several studies have reported the effect of positive margins for tumor cells on the rate of local recurrence of cancer for in situ and invasive disease. In 2002, Singletary reviewed 10 studies, with more than 4000 patients of early invasive cancer with stages I and II diseases [22]. The recurrence rates ranged from 8 to 25% (mean 16%) for positive margins, whereas, for negative margins, the recurrence rates varied between 2 and 7% (mean 4%). They also defined the positive margin as tumor cells touching the cut margin of the specimen. The negative margin ranged between margin widths of 1–3 mm.

There exists significant variation in perception of surgical margins not only amongst various centers but also between the multidisciplinary teams, including surgeons, pathologists, and radiologists involved in the management of the disease [23]. The debate is over the negative margin definition and the exact margin width to describe negative margins [10].

Houssami et al. found an impact of surgical margins therapy for invasive breast meta-analysis, based on 21 studies, on the local recurrence in breast conservation cancer. They emphasized further the high recurrence rate with positive margin status. They also highlighted the lack of consensus regarding the margin width. They concluded that the adoption of wider margins (e.g., 5 mm) was unlikely to have a substantial additional benefit for long-term local control over using a narrow margin width (i.e., 1-2 mm) for declaring negative margins in invasive breast cancer [13].

Only recently, some regional guidelines have come to support the concept of “no tumor at inked margin” to be taken as negative margin [12]. The practice varies worldwide including Pakistan [24, 25]. Furthermore, for in situ cancer, the least acceptable margin described is 2 mm [26] and as high as a 5 mm margin is taken as acceptable negative margin in some regions [24]. Hence, shrinkage of margin after formalin fixation may still hold strong implications on further treatment strategy of breast cancer.

Although margin shrinkage is much more relevant for BCS specimen, as far as implications are concerned, our study population at that particular cross-section of time underwent mastectomy mostly due to the reasons already described above. It was not possible for us to complete the needed sample size if we took BCT specimen only. Hence, we aimed to describe shrinkage of tumor-free margin after formalin fixation affecting both types of specimen, that is, mastectomy and BCT. This can be taken as one of the limiting factors of our study.

Formalin fixation of surgical specimen has been described in various types of tumors as being responsible for shrinkage of tumor specimen and its tumor-free margins. The various tumors that have been studied include colorectal, esophageal, skin, and, more recently, breast tumors [15, 16, 27, 28]. The comparison of preformalin fixation macroscopic margin and postformalin fixation microscopic margin in principle may not seem compatible; however, it is the most convenient and practical way. This method has already been described in assessing esophageal and colorectal as well as breast tumors in the past [27, 28].

Our study showed shrinkage of margins after formalin fixation. This finding has also been found in another study by Yeap et al. The study confirms a striking shrinkage of tumor-free margin in their specimen with a mean reduction of 3.5 mm which was statistically significant [15]. Another study by Krekel et al. [17], however, contradicts these results and does not show any shrinkage after formalin fixation in sixty-eight breast specimens.

In order to see the impact of variables like age, tumor type, tumor grade, tumor stage, and type of surgery on the margin shrinkage, stratification was further done, which failed to add much to the inference. What seemed prominent was the fact that the shrinkage was slightly less for carcinoma in situ and breast conservation surgery specimens. This may have been a result of a small sample size of these two subsets of our study population.

There were some other limitations to our study as well. Firstly, it was a cross-sectional study due to which long-term outcome in the form of recurrence could not be analyzed. Secondly, the mastectomy specimens in our study had only one margin to be described, which was the posterior tumor-free margin, even though all margins are equally important in predicting local recurrence. We used the closest tumor-free margin as an accurate representative of the shrinkage of the rest of the margins as well.

However, our research gives us several directions for the future. Firstly, it can facilitate the journey towards establishing a universal definition of margin analysis. There is also a need for more research on the techniques of transport and fixation of specimen to avoid handling errors. We would also like to suggest that more research be conducted on the effect of formalin fixation, preferably on a larger sample size and on BCT specimen only for better inference. Prospective randomized control trials can also be considered to compare the recurrence of tissue analyzed with and without formalin fixation.

## 5. Conclusion

Our breast cancer specimens show considerable shrinkage of their tumor-free surgical margins due to tissue fixation with formalin. This inference may have effects on the postoperative management plan of these patients and can end up in decisions for further surgery or adjuvant radiotherapy. However, further research is needed to solidify our claim.

## Figures and Tables

**Table 1:** Demographic data.

Variable	Number of patients (*n* = 100)
Age	
<40 years	18 (18%)
41–60 years	55 (55%)
>61 years	27 (27%)
Type of surgery	
Mastectomy	90 (90%)
Simple mastectomy	39
Modified radical mastectomy	51
Breast conservation surgery	10 (10%)
Wide local Excision	9
Quadrantectomy	1
Cancer type	
Infiltrating ductal carcinoma (IDC)	82
Lobular carcinoma	6
Metaplastic carcinoma	1
Micropapillary carcinoma	1
Apocrine carcinoma	2
Ductal carcinoma in situ	8
Stage	
0 (DCIS)	8
I	18
II	51
III	23
Grade of tumor∗ (*n* = 92)	
I	5
II	65
III	23

*indicates that the grade of tumor applied to invasive tumors only.

**Table 2:** Analysis of specific margins.

	Prefixation margins	Postfixation margins	Difference (postfixation − prefixation)
Mean closest tumor-free margin (mm)	22.43	20.28	2.14 (95% C.I. = 1.77 − 2.52)

**Table 3:** Stratification of variables.

Variables	Categories	Mean margin reduction (±S.D.)
Age	≤40 years	2.12 mm ± 2.08 mm
	41–60 years	2.17 mm ± 1.68 mm
	≥61 years	2.11 mm ± 2.18 mm

Type of surgery	Mastectomy	2.21 mm ± 1.91 mm
	Breast conservation surgery	1.60 mm ± 1.50 mm

Type of tumor	Invasive cancer	2.18 mm ± 1.69 mm
	In situ cancer	1.68 mm ± 1.71 mm

Stage	0	1.68 mm ± 1.71 mm
	I	2.56 mm ± 1.9 mm
	II	2.23 mm ± 1.9 mm
	III	1.7 mm ± 1.9 mm

Grade	I	2.3 mm ± 1.7 mm
	II	2.1 mm ± 1.82 mm
	III	2.13 mm ± 2.15 mm
